# Mucinous cystic neoplasm of the liver with biliary communication: a great imitator, diagnostic dilemma and surgical challenge

**DOI:** 10.1093/jscr/rjag137

**Published:** 2026-03-12

**Authors:** Elizabeth M Hines, Jack Butler, Lovell Aseervatham

**Affiliations:** Department of General Surgery, Sunshine Coast University Hospital, 6 Doherty St Birtinya, Brisbane, QLD 4575, Australia; Pathology Queensland, Anatomical Pathology, Sunshine Coast University Hospital, 6 Doherty St Birtinya, Brisbane, QLD 4575, Australia; Department of General Surgery, Sunshine Coast University Hospital, 6 Doherty St Birtinya, Brisbane, QLD 4575, Australia

**Keywords:** mucinous cystic neoplasm of the liver (MCN-L), cystic liver lesions, hepatobiliary surgery, intraductal papillary neoplasms of the bile duct (IPN-B), hydatid cyst, misdiagnosis, hepatectomy, pericystectomy

## Abstract

Mucinous cystic neoplasms of the liver (MCN-L) are rare tumours. We report a challenging case initially characterized as hydatid cyst. The patient underwent initial pericystectomy converted to completion left hepatectomy for definitive management. Histopathology confirmed MCN-L with focal high-grade dysplasia. Crucially, the resection specimen revealed a previously unrecognized finding: direct cyst rupture into the biliary tree, associated with bile encrustation, calculi formation, and inflammation. This case highlights that MCN-L can clinically and radiologically mimics other cystic liver lesions, necessitating histopathology for definitive diagnosis. The phenomenon of biliary communication and its sequelae are not readily accounted for in the current World Health Organization essential diagnostic criteria for MCN-L. Such cases may warrant the allowance of biliary communication within diagnostic criteria, to better reflect the spectrum of this lesion and avoid diagnostic pitfalls. Complete surgical excision is the mainstay of therapy, with long-term surveillance being essential, particularly in neoplasms with high-grade dysplasia.

## Introduction

Mucinous cystic neoplasms of the liver (MCN-L) are exceptionally rare cyst-forming epithelial neoplasms with malignant potential; comprising <5% of all cystic liver lesions, which themselves affect only 5%–10% of people worldwide [1, 2]. The 2019 World Health Organization (WHO) 5^th^ edition classification of tumours differentiates MCN-L from intraductal papillary neoplasm of the bile duct (IPN-B) by the presence of hormone receptor positive ovarian-like stroma and absence of bile duct involvement [2]. Conversely, there are a handful of cases documenting MCN-L with biliary tree communication, [1, 3, 4] or intact polypoid projection/prolapse into bile duct lumens [5–7] highlighting a non-comprehensive gap in the current WHO classification diagnostic criteria [1–4].

MCN-L predominantly affects women in the fifth decade of life, is often asymptomatic but can present with abdominal pain/distention and/or obstructive jaundice secondary to mass effect, and patients often lack pre-existing/concomitant liver disease [4]. On imaging MCN-L favours the left lobe of the liver (segment IV), appearing as a multiloculated cyst with internal septations, with an indolent growth pattern [6]. Because of rarity, inconsistent clinical symptoms, lack of specific biochemical markers, and high variegation of imaging patterns, MCN-L is often hard to distinguish from other cystic diseases resulting in a high rate of misdiagnoses [6]. Specifically, MCN-L has been frequently documented to imitate and be mistaken for hydatid cysts [8].

## Case report

A Caucasian female smoker in her mid 40’s with no other relevant medical history was referred to our hepatobiliary surgical service after routine blood tests revealed abnormal liver functions (Alanine aminotransferase 187 U/L, Aspartate aminotransferase 138 U/L, and Gamma-glutamyl transferase 253 U/L). Patient consent was obtained for the deidentified publication of imaging and histopathology. She was asymptomatic, reporting no history of abdominal pain, weight loss, or constitutional features. There was no history of travel to hydatid endemic regions, use of hepatotoxic medications or exposure to farm animals.

**Figure 1:**
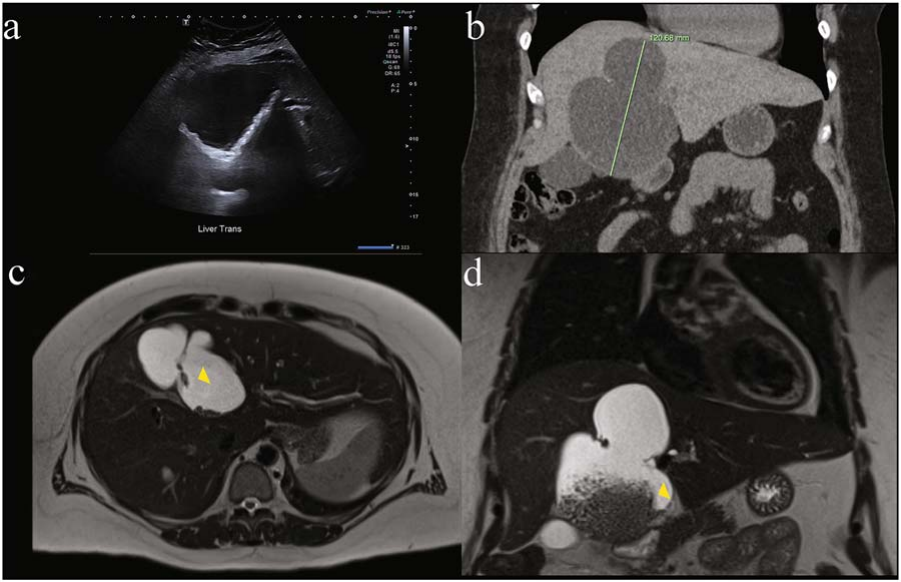
Diagnostic difficulty represented in single patient imaging series of mucinous cystic neoplasm of the liver (MCN-L). There is wide variation between modalities in same patient: (a) ultrasound of liver demonstrating internal matrix echogenicity or debris, (b) coronal contrast enhanced CT showing non-enhancing cystic lesion 12.1 cm in largest dimension occupying most of segment IV a and b with internal debris, peripheral calcifications and left hepatic lobe biliary tree dilatation reported as concerning for biliary cystadenoma, and magnetic resonance imaging (MRI) T2-weighted. (c) Axial and (d) coronal views showing dependent debris with characteristic ‘hydatid sand’ (yellow arrowhead) appearance and septations concerning for hydatid disease that were later found to be bile stones.

Ultrasound measured a 13 × 17 × 9.5 cm complex cystic lesion in segment IVb of the liver, exhibiting thick septations, wall calcifications with internal matrix echogenicity or debris, without common bile duct (CBD) dilatation (5.3 mm) (Fig. 1a). Subsequent computed tomography (CT) characterized the lesion as a large (9.8 × 6.8 × 12.1 cm) non-enhancing cyst occupying segments IV a/b with internal debris, peripheral calcifications and left hepatic lobe biliary tree dilatation concerning for biliary cystadenoma (Fig. 1b). Magnetic resonance (MR) demonstrated dependent debris with characteristic "hydatid sand" appearance and septations (Fig. 1c and d), without biliary involvement or dilatation. Despite negative hydatid serologies, WHO CE1 and CE2 active stage classification according to WHO informal working groups on Echinococcosis guidelines (constructed for ultrasound) was suggested. Consequently, preoperative albendazole 400 mg twice daily was commenced on the advice of the infectious disease specialists. Tumour markers Alpha-fetoprotein (AFP) and Carcinoembryonic antigen (CEA) were not elevated. Ca 19–9 was not tested.

Pericystectomy, was performed with cholecystectomy via rooftop incision extended along the linea alba to the xiphisternum. The cyst had no obvious communication with the biliary tree. When inadvertently ruptured, sited granular contents resembled hydatid sand. During parenchymal dissection, significant bleeding from the middle hepatic vein caused the patient to behave in a coagulopathic manner, necessitating temporization with saline packing and laparostomy with immediate recovery to the ICU for physiological correction whilst intubated. A completion left hepatectomy and cholangiogram were performed the following day. Persistently elevated biliary serum markers necessitated an endoscopic retrograde cholangiopancreatogram immediately post-surgery for sphincterotomy and CBD stenting.

**Figure 2:**
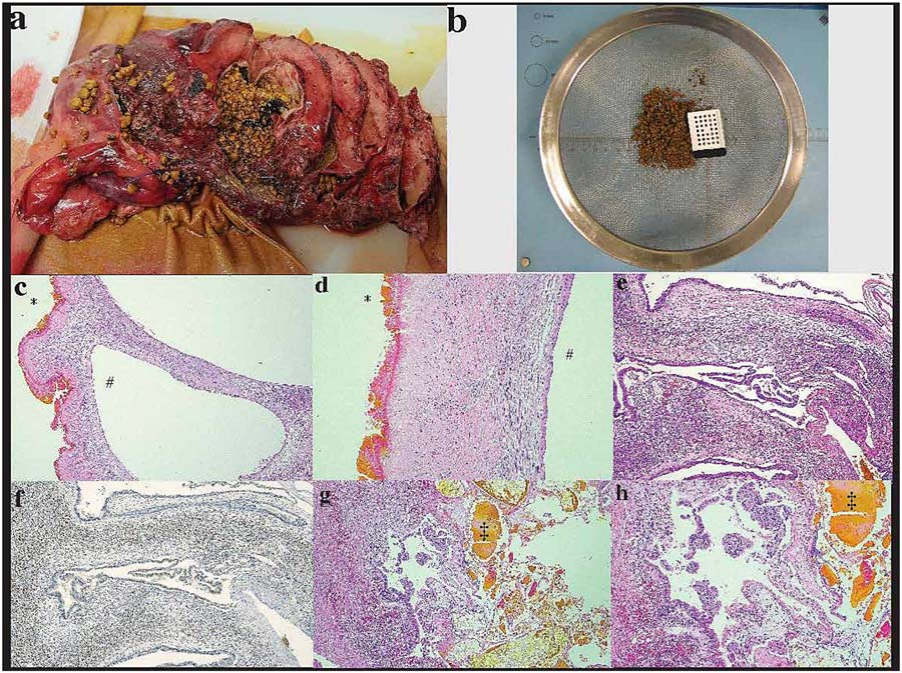
Histopathologic examination: (a and b) initial sectioning for fixation showed a large multilocular cystic lesion with numerous tan calculi and biliary debris in the lumen, (c and d) the internal surface of the largest locule predominantly had a green-tinged roughened texture, corresponding to microscopic bile encrustation and denuded epithelium (*) with sclerosis of the cyst wall. The intact epithelium, well preserved in adjacent smaller locules (#), was cuboidal to columnar mucinous epithelium with (e) ovarian-like subepithelial stroma exhibiting strong positivity for (f) PR immunohistochemistry. (g and h) There was focal luminal suppurative inflammation and biliary calculi(‡)/debris seen microscopically as well as focal high-grade dysplasia and papillary tufting. No invasive component was present**.**

Histopathologic examination showed numerous 2 – 7 mm tan calculi present within the lumina of the multicystic lesion (Fig. 2a and b). Initial representative sections on microscopy demonstrated multilocular cystic spaces with predominantly denuded epithelium, marked bile encrustation on the luminal aspect and sclerosis of the cyst wall (Fig. 2c and d). There was luminal suppurative inflammation along with acute inflammation and foreign-body giant cell reaction in the cyst wall. The remnant intact epithelium was cuboidal to columnar with low-grade dysplasia and subepithelial ovarian-like stroma (OLS) that was strongly positive for progesterone receptor (PR) (Fig. 2e and f) and weakly positive for estrogen receptor (ER) immunohistochemistry. These features confirmed the diagnosis of MCN-L. Microscopic examination of the entire cyst wall to exclude associated invasive carcinoma showed focal papillary projections with high-grade dysplasia (Fig. 2g and h)—no invasion was present. Overall, the MCN-L showed extensive reactive changes, possibly secondary to rupture into native bile ducts with resulting fibrosis, inflammation, bile encrustation, and calculi formation. These reactive changes simultaneously diminished and obscured the diagnostic features of the underlying MCN-L.

The background liver parenchyma showed moderate steatosis without any other significant pathology. Sections including the larger intra-hepatic bile duct branches showed dilatation with focally denuded epithelium. Where present, residual epithelium within these ducts showed focal reactive appearing atypia and intestinal metaplasia. These changes adjacent to the lesion were again considered consistent with possible previous rupture of the MCN-L. Notably the gallbladder showed no calculi - only mild fibromuscular hyperplasia and chronic inflammation of the gallbladder wall.

## Discussion

To our knowledge this is the first case demonstrating histopathological evidence of bile encrustation including calculi formation within the lumen of the cyst. It was further complicated by the presence of focal intestinal metaplasia and focal papillary architecture blurring the boundary between MCN-L (OLS-present, no biliary communication) and the differential diagnosis of IPN-B (biliary communication, no OLS). Despite these unusual features, the definitive presence of OLS confirmed the diagnosis of MCN-L. This case, alongside others, suggests that an allowance for biliary tract communication in the WHO essential diagnostic criteria may be necessary to reflect these rare cases and avoid diagnostic pitfalls when other features may also be diminished. Such an allowance in essential diagnostic criteria would also align with the current, less explicit, WHO definition of MCN ‘typically showing no communication with the bile ducts’.

Clinically, this has significant implications, as misdiagnosis based on overlapping imaging features and non-specific biomarkers may lead to inappropriate preoperative management. Given the risk of recurrence and malignant transformation, complete surgical resection remains the gold standard. Future research should investigate whether biliary involvement correlates with distinct molecular profiles or clinical outcomes, ultimately refining diagnostic precision and optimizing patient management. Lastly, we advocate for routine multidisciplinary evaluation of such cases to optimize diagnostic accuracy and patient outcomes.

## References

[1] Ferreira R, Abreu P, Jeismann VB et al. Mucinous cystic neoplasm of the liver with biliary communication: case report and surgical therapeutic option. BMC Surg 2020;20:328. 10.1186/s12893-020-01003-3 33308210 PMC7733287

[2] Nagtegaal ID, Odze RD, Klimstra D et al. The 2019 WHO classification of tumours of the digestive system. Histopathology 2020;76:182–8. 10.1111/his.13975 31433515 PMC7003895

[3] Anand S, Chandrasekar S, Raja K et al. Mucinous cystic neoplasm of the liver with biliary communication: an exception to the current classification. BMJ Case Rep 2019;12:bcr-2018-227063. 10.1136/bcr-2018-227063 PMC6340565 30635308

[4] Rodriguez RM, Barrio M, Parker ML et al. Mucinous cystic neoplasms of the liver: presence of biliary communication. J Surg Case Rep 2019;2019:rjz364. 10.1093/jscr/rjz364 31832138 PMC6900340

[5] Takano Y, Nagahama M, Yamamura E et al. Prolapse into the bile duct and expansive growth is characteristic behavior of mucinous cystic neoplasm of the liver: report of two cases and review of the literature. Clin J Gastroenterol 2015;8:148–55. 10.1007/s12328-015-0569-8 25951998 PMC4481294

[6] Fukui Y, Murata A, Shimizu S et al. Mucinous cystic neoplasm of the liver with polypoid nodule prolapsing into the bile duct: a case report and review of literature. Surg Case Rep 2022;8:177. 10.1186/s40792-022-01511-9 36138328 PMC9500141

[7] Pattarapuntakul T, Ovartlarnporn B, Sottisuporn J. Mucinous cystic neoplasm of the liver with extrahepatic growth presenting with ascending cholangitis diagnosed by endoscopic ultrasound features: a case report. J Med Case Reports 2018;12:33. 10.1186/s13256-017-1560-4 PMC5813420 29444709

[8] Soni S, Pareek P, Narayan S et al. Mucinous cystic neoplasm of the liver (MCN-L): a rare presentation and review of the literature. Med Pharm Rep 2021;94:366–71. 34430860 10.15386/mpr-1543 PMC8357353

